# Honeybees are buffered against undernourishment during larval stages

**DOI:** 10.3389/finsc.2022.951317

**Published:** 2022-11-18

**Authors:** Felix Schilcher, Lioba Hilsmann, Markus J. Ankenbrand, Markus Krischke, Martin J. Mueller, Ingolf Steffan-Dewenter, Ricarda Scheiner

**Affiliations:** ^1^ Behavioral Physiology and Sociobiology, Theodor-Boveri-Institute, Biocenter, Julius-Maximilians-Universität Würzburg, Würzburg, Germany; ^2^ Center for Computational and Theoretical Biology (CCTB), Julius-Maximilians-Universität Würzburg, Würzburg, Germany; ^3^ Julius-von-Sachs-Institute of Biosciences, Theodor-Boveri-Institute, Biocenter, Julius-Maximilians-Universität Würzburg, Würzburg, Germany; ^4^ Animal Ecology and Tropical Biology, Theodor-Boveri-Institute, Biocenter, Julius-Maximilians-Universität Würzburg, Würzburg, Germany

**Keywords:** nutrition, *in-vitro* rearing, juvenile hormone, nurse bees, foragers, triglycerides, undernourishment, task allocation

## Abstract

The negative impact of juvenile undernourishment on adult behavior has been well reported for vertebrates, but relatively little is known about invertebrates. In honeybees, nutrition has long been known to affect task performance and timing of behavioral transitions. Whether and how a dietary restriction during larval development affects the task performance of adult honeybees is largely unknown. We raised honeybees *in-vitro*, varying the amount of a standardized diet (150 µl, 160 µl, 180 µl in total). Emerging adults were marked and inserted into established colonies. Behavioral performance of nurse bees and foragers was investigated and physiological factors known to be involved in the regulation of social organization were quantified. Surprisingly, adult honeybees raised under different feeding regimes did not differ in any of the behaviors observed. No differences were observed in physiological parameters apart from weight. Honeybees were lighter when undernourished (150 µl), while they were heavier under the overfed treatment (180 µl) compared to the control group raised under a normal diet (160 µl). These data suggest that dietary restrictions during larval development do not affect task performance or physiology in this social insect despite producing clear effects on adult weight. We speculate that possible effects of larval undernourishment might be compensated during the early period of adult life.

## Introduction

Malnourishment has long been a topic of research when it comes to human development, especially when observing malnourished children in third world countries. Early malnourishment can have severe cognitive, developmental and behavioral effects (1, 2). However, little is known about long-term effects of malnourishment in insects. Wild bee abundance and diversity is decreasing (3), due in part to poor nutritional landscapes (4). The honeybee is an ideal model organism to study effects of malnourishment on behavior for the diversity of methods available and to serve as a proxy for effects of malnutrition on wild bees (5). Honeybee colonies are highly complex superorganisms that depend on the proper execution and timing of tasks by their members (6, 7). Apart from reproduction, all colony tasks are executed by sterile female workers. Young honeybee workers perform in-hive tasks, like nursing and cleaning, while older honeybees work at the periphery of the hive until they eventually leave the hive to forage for resources. However, this temporal polyethism can be accelerated, halted or even reversed (8). For example, depleting a honeybee colony of foragers can lead to an increase in precocious foragers (9). Nutrition plays an important role in temporal polyethism. Starvation of honeybee colonies can lead to an increase in foragers compared to well-fed colonies (10). Furthermore, nurse bees have significantly higher triglyceride (TG) levels compared to foragers, leading to the conclusion that a reduction in lipids might accelerate the transition to foragers (11). In support of this assumption, low TG levels were reported in precocious foragers and high TG levels in foragers reverting to nursing tasks, thereby dissociating task from age (11). Later experiments showed a direct link between a reduction of lipids and an increase in foragers. Feeding honeybees with 5-tetradecyloxy-2-furanocarboxylic acid (TOFA), while simultaneously restricting pollen consumption decreased honeybee TG levels and increased foraging activity (12). This leads to the conclusion that lipid levels as well as food quantity seem to be an important factor in honeybee temporal polyethism. However, food quality also seems to be of high importance. Newly emerged honeybees feeding on sugar syrup show increased gene expression of two genes involved in juvenile hormone (JH) synthesis compared to newly emerged honeybees feeding on beebread (13). Juvenile hormone is known to be an important factor in the transition from nursing to foraging. Treating honeybees with the JH analog methoprene started and stopped nursing tasks earlier compared to the control (14) and led to an earlier initiation of foraging. Kaatz etal. (15) showed that starving honeybees increases JH production in foragers and even more so in nurse bees. Nurse bees generally have lower titers of JH than foragers but higher titers of vitellogenin (16–18). This egg yolk precursor protein is generally assumed to be the suppressor of JH, with JH possibly also suppressing vitellogenin (VG) by hitherto unknown mechanisms (19). Once the suppressor VG is used up by nurse bees for producing brood food, JH titers can increase and induce the nurse-forager transition. This transition coincides with an increase in sucrose responsiveness which can serve as a behavioral indicator (20–23). Furthermore, pollen consumptions is linked to higher VG titers (24, 25). While no link has been found in honeybees, starvation is known to decrease VG levels in *Romalea microptera* (26). However, starvation does not only affect adult honeybees but also their larvae. Wang etal. (27) showed that starving honeybee larvae leads to increased JH titers in newly emerged workers and in seven-day-old worker bees, thus linking JH titers to larval starvation. While little is known about larval starvation on adult honeybee behavior, the importance of the quality of larval nutrition is. High carbohydrate diets are known to increase the resting metabolic rate and survivability in honeybees (28), while solitary bee larvae are known to prioritize carbohydrates over protein (29).

In this study, we reared honeybees *in-vitro* under different diets and analyzed behavioral and physiological parameters in addition to weight, which is commonly analyzed regarding honeybee starvation. We here test the hypothesis that undernourishment during larval development induces a precocious increase in JH titers in young adult worker bees, resulting in an earlier onset of foraging.

## Material and methods

### *In-vitro* rearing

To acquire honeybee larvae, the queens were caged for 24h. After three days the newly emerged honeybee larvae were transferred into the laboratory and reared *in-vitro* according to a standardized protocol (30–36). Three groups of honeybees were reared under different diets [**Table 1**: 150 µl (“undernourished”), 160 µl (“normal diet”) and 180 µl (“overfed”)] based on data of (37). Food was provided on six consecutive days according to **Table 1** (37). On day 1, honeybees received diet A, on day 3 they received diet B and on the following days honeybees received diet C. Food quality did not differ between the treatments, just the quantity differed. Honeybees were reared according to (36). In short, age-controlled larvae were individually grafted and placed into small plastic cups (Weisel cups, Heinrich Holtermann KG, Brockel, Germany). These cups were transferred into 48-well plates and maintained in an incubator at 35°C and 95% relative humidity (RH) over six days. Larval food contained royal jelly, fructose, glucose, yeast and water according to **Table 2**. After pupation, the pupae were placed into fresh 48- well plates, transferred into a new incubator and maintained at 35°C and 75% RH and left untouched until emergence, apart from sparse mortality checkups. In total, about 75% of all larvae were reared successfully into adult honeybees. No changes could be observed in rearing success between the three diets (χ ^2^ test: χ = 3.594, df = 2, p = 0.1658; data not shown).

**Table 1:** Different feeding regimes for the three treatment groups.

Treatment	Day 1Diet A	Day 2	Day 3Diet B	Day 4Diet C	Day 5Diet C	Day 6Diet C
150 µl (undernourished)	20 µl	X	20 µl	30 µl	40 µl	40 µl
160 µl (normal diet)	20 µl	X	20 µl	30 µl	40 µl	50 µl
180 µl (overfed)	20 µl	X	20 µl	30 µl	50 µl	60 µl

Changes in food quantity accrued only on the 5^th^ and 6^th^ days of feeding according to (37).

**Table 2:** Standard larval diet according to (30).

Diet A	Royal Jelly	Fructose	Glucose	Yeast	Water
[%]	50	6	6	1	37
[g]	20	2.4	2.4	0.4	14.8
**Diet B**	**Royal Jelly**	**Fructose**	**Glucose**	**Yeast**	**Water**
[%]	50	7.5	7.5	1.5	33.5
[g]	20	3	3	0.6	13.4
**Diet C**	**Royal Jelly**	**Fructose**	**Glucose**	**Yeast**	**Water**
[%]	50	9	9	2	30
[g]	20	3.6	3.6	0.8	12

All treatment groups received the same diets with variations in total food volume.

### Behavioral experiments

For the nursing behavior observations, emerging honeybees were marked using colored number plates (Opalith Classic Garnitur; Heinrich Holtermann KG; Germany) and superglue (UHU^®^ Sekundenkleber blitzschnell Pipette; UHU GmbH & Co. KG; Germany). Afterwards, they were transferred into cages (internal dimensions: 8 cm x 5 cm x 5 cm; three impenetrable- & one wire framed wall) and were fed *ad libitum* with pollen, tap water and 50% sugar water. After a night in an incubator maintained at 35°C and 50% RH for the superglue to fully dry, the honeybees were integrated into a four-frame observation hive. For this purpose, a funnel was used to insert the newly emerged honeybees into the hive. To increase acceptance of the host colony, the newly emerged honeybees were sprayed sparingly with thyme extract (Thymiangeist; Heinrich Holtermann KG; Germany). One day after the integration, the observations began by removing one outside wall of the observation hive. Thus, the experimenter could observe the honeybees through a see-through Plexiglas wall. Observations were conducted each day from 10:30 a.m. until 2:30 p.m. for four consecutive weeks. All four frames were scanned systematically in a pseudo-randomized order, recording every visible honeybee with its head in an open brood cell for at least 15 s (38).

For the foraging behavior observations, honeybees were treated identically as before. However, instead of colored number plates, radio-frequency identification (RFID) tags (mic3-TAG 64bit read only, carrier frequency: 13.56 MHz, microsensys GmbH, Erfurt, Germany) were used to mark the emerging honeybees (36, 39). After the drying period in the incubator, the cages were placed into four six-frame queen-right mini plus colonies (small, standardized colonies containing approx. 3,000 bees) outfitted with two specifically designed scanners (MAJA Bundle Bee Identification System: iID 2000 ISO 15693 optimized, Micro-Sensys GmbH). Both scanners were placed in front of the hive entrance and were distinguishable by a unique number. Honeybees leaving from or returning to the colony had to pass both scanners in a defined order. Data was acquired as established previously (39). Cages were opened after one day, honeybees were sprayed sparingly with thyme extract, and the marked honeybees were able to move about freely in the colony, while the recordings began. This adaptation period of one day was used to increase acceptance of the young honeybees once they had been released into the hive.

#### Observed behaviors

For the nursing observations, multiple parameters were observed. Onset and termination of nursing were defined as the first and last days a marked honeybee was recorded with its head in a brood cell. Nursing span was defined as the difference between onset and termination of nursing plus one.

Foraging observations were conducted in the same manner as done before (39), observing the onset and termination of foraging in addition to the duration of a foraging trip. Additionally, the foraging span and foraging trips per bee per day were recorded. Foraging span was defined as above, as the difference between onset and termination of foraging plus one.

### Weight, juvenile hormone, triglycerides and sucrose responsiveness

On the second day of the experiment and 7, 14 and 21 days afterwards, five honeybees were removed from each colony and treatment to perform further analyses. First, honeybees were immobilized on ice and weighted (36). Then they were fixed in metal tubes and fed until satiation using 30% sugar water (40, 41). After one hour of adjustment, sucrose responsiveness was quantified using the proboscis extension response (PER) assay (21, 22). First, the antennae of each bee were touched with water. Afterwards, they were sequentially touched with increasing sucrose concentrations of equal logarithmic distance (0.1% sucrose, 0.3% sucrose, 1% sucrose, 3% sucrose, 10% sucrose and 30% sucrose) with an intertrial interval of 2 min to avoid intrinsic sensitization (40, 41). The occurrence of proboscis extension was recorded for each stimulation of the antennae. The sum of the seven PER responses including water of an individual honeybee represents the gustatory response score (GRS) as established previously (20, 21).

After quantifying individual sucrose responsiveness, honeybees of the different feeding regimes were immobilized on ice for a second time and fixed with needles onto a Styrofoam plate. We extracted 5 µl of hemolymph by piercing the cuticle in between the fourth and fifth abdominal segments using glass micro capillaries (servoprax^®^, A1 0115; servoprax GmbH; Germany). Hemolymph was stored at -80°C until analyzation. Levels of hemolymph JH were analyzed by LC‐MS/MS using a Waters Acuity ultrahigh‐performance liquid chromatography system coupled to a Waters Micromass *Quattro Premier* triple quadrupole mass spectrometer (Milford, MA) as described before (42). After the hemolymph extraction, the honeybees were frozen in liquid nitrogen and half of their fat bodies was crushed in a cooled mixer mill (MM 400; Retsch) using zirconia beads. The fat bodies were dissected by opening the abdomen and removing the digestive tract, the sting, the tracheal tissue and the ventral nerve cord. The resulting fat body was halved using dissection scissors to get two halves of a fat body with the approximately the same weight. Later statistical analysis were controlled for weight of the corresponding fat body halves. Afterwards, the triglycerides were extracted twice using chloroform (1 ml), methanol (0.5 ml) and two triacylglycerol (TAG) standards (2.5 µg each, 10:0 TAG & 17:0 TAG). After mixing and centrifugation, the supernatant was collected and 0.88% aqueous KCl (0.75 ml) was added. The upper phase was discarded and 0.25 ml methanol and 0.25 ml H_2_O were added to the lower phase containing the lipid extract. Afterwards, the lower phase was dried under reduced pressure using a rotational vacuum concentrator (RVC 2-25 CDplus; CHRIST) at 50°C. The dried residue was dissolved in 100 µl isopropanol and frozen at -20°C until analysis with a UPLC–qTOF-MS (Synapt G2 HDMS; Waters) as described in (43). The data was analyzed using MassLynx™ software from Waters^®^. Only the ten most frequently appearing triglycerides (TGs) were selected for statistical analysis as they represent more than 80% of all TGs (**Supplement Figure 1**).

### Statistics

Statistical analyses were conducted using R (4.1.2). and the R packages “glmm TMB” V. 1.1.2.3 (44), “lme4” V. 1.1-2s7.1 (45), “DHARMa” V. 0.4.4 (46), “rstatix” V 0.7.0 (47), “ggeffects” V. 1.1.1 (48), “emmeans” V. 1.7.0 (49), “reshape2” V 1.4.4 (50), “tidyverse” V. 1.3.1 (51), “dplyr” V. 1.0.7 (52) and “Rmisc” V. 1.5 (53). A Shapiro-Wilk test was used to test the data for normal distribution. Since data was not normally distributed most of the time, probability data was analyzed with a general linear model (GLM) and effects of larval nutrition on task performance and physiology were investigated with a generalized linear mixed model (GLMM). For the experiment studying nursing behavior, larval nutrition was used as a fixed factor. For the experiment on foraging behavior, larval nutrition was used as a fixed factor and the four different colonies were inserted into the model as a random factor. The family (**Tables 3**–**6**) was chosen according to the best fit in a DHARMa residual analysis (46). Physiological data was handled in the same way as the foraging data. *Post-hoc* analyses were conducted using Tukey multiple comparison tests. Graphs were constructed using R (4.1.2). and the R packages “ggplot2” V 3.35 (54), “cowplot” V 1.1.1 (55) and “ggpubr” V. 0.4.0 (56).

**Table 3:** Test statistics for the analysis conducted in **Figures 1A**, **2A**.

Analysis	Figure	Treatment	Sample size	Test	Predicted	95% CIlower CI – upper CI
Nursing probability	1A	150 µl160 µl180 µl	195192180	GLMfamily = binominal	0.3640.3230.256	0.3 – 0.4340.261 – 0.3920.197 – 0.324
				Contrasts150 µl vs 160 µl150 µl vs 180 µl160 µl vs 180 µl	Odds ratio1.201.671.39	Significancep = 0.6702p = 0.0619p = 0.3265
Foraging probability	2A	150 µl160 µl180 µl	101210051025	GLMfamily = binominal	0.1350.1620.164	0.116 – 0.158 0.142 – 0.1887 0.142 – 0.188
				Contrasts150 µl vs 160 µl150 µl vs 180 µl160 µl vs 180 µl	Odds ratio0.8030.7990.995	Significancep = 0.1866p = 0.1689p = 0.9989

Predicted probabilities and the 95% confidence interval (CI) of the GLM analysis for the probability of honeybees to perform nursing or foraging tasks. Odds ratios and significance levels of the Tukey *post hoc* analysis are also shown.

**Table 4:** Test statistics for the analysis conducted in **Figures 1B–D**.

Analysis	Figure	Treatment	Sample size	Test	Predicted	95% CIlower CI – upper CI
Onset of nursing [days]	1B	150 µl 160 µl 180 µl	70 62 46	GLMM family = compois link = log	4.79 4.94 5.00	4.46 – 5.14 4.58 – 5.31 4.59 – 5.44
				ANOVA: χ = 0.6808, p = 0.7155		
Termination of nursing [days]	1C	150 µl 160 µl 180 µl	70 62 46	GLMM family = compois link = log	5.14 5.55 5.36	4.75 – 5.53 5.13 – 5.96 4.87 – 5.84
				ANOVA: χ = 1.9503, p = 0.3771		
Nursing span [days]	1D	150 µl 160 µl 180 µl	70 62 46	GLMM family = nbinom2 link = log	1.35 1.61 1.46	1-11 – 1-65 1-33 – 1-96 1-15 – 1-85
				ANOVA: χ = 1.5224, p = 0.4671		

Predicted means and the 95% confidence interval (CI) of the GLMM (including chosen family and link) analysis as well as the conducted ANOVA for the onset of nursing, termination of nursing and the nursing span. Results of the *post hoc* analysis are shown in **Supplementary Table 1**.

**Table 5:** Test statistics for the analysis conducted in **Figures 2B-F**.

Analysis	Figure	Treatment	Sample size	Test	Predicted	95% CIlower CI – upper CI
Onset of foraging [days]	2B	150 µl 160 µl 180 µl	137 164 168	GLMM family = nbinom1 link = log	9.85 9.75 9.91	9.26 – 10.47 9.21 – 10.31 9.38 – 10.47
				ANOVA: χ = 0.1728, p = 0.9172		
Termination of foraging [days]	2C	150 µl 160 µl 180 µl	137 164 168	GLMMfamily = nbinom1 link = log	13.94 14.04 14.14	12.92 – 15.05 13.09 – 15.05 13.20 – 15.14
				ANOVA: χ = 0.0709, p = 0.9652		
Foraging span [days]	2D	150 µl 160 µl 180 µl	137 164 168	GLMMfamily = nbinom1 link = log	5.10 5.35 5.17	4.4 – 5.9 4.69 – 6.11 4.52 – 5.9
				ANOVA: χ = 0.2829, p = 0.8681		
Duration per foraging trip [min]	2E	150 µl 160 µl 180 µl	137 164 168	GLMMfamily = nbinom1 link = log	21.87 23.74 22.54	19.15 – 24.99 21.06 – 26.75 19.97 – 25.44
				ANOVA: χ = 0.9792, p = 0.6129		
Foraging trips per day	2F	150 µl 160 µl 180 µl	137 164 168	GLMMfamily = nbinom1 link = log	2.02 2.11 2.06	1.82 – 2.24 1.91 – 2.31 1.87 – 2.26
				ANOVA: χ = 0.3538, p = 0.8379		

Predicted means and the 95% confidence interval (CI) of the GLMM (including chosen family and link) analysis as well as the conducted ANOVA for the onset of foraging, termination of foraging, foraging span, duration per foraging trip and foraging trips per day. Results of the *post hoc* analysis are shown in **Supplementary Table 2**.

## Results

### Behavioral experiments

**Figure 1:**
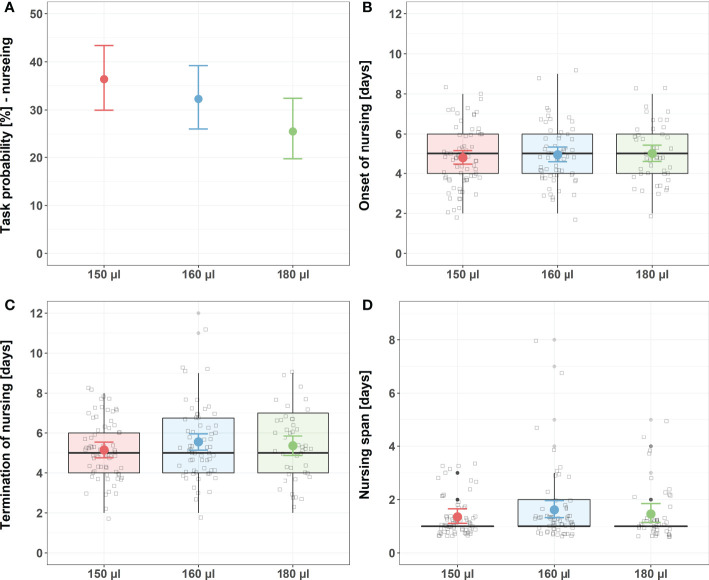
Influence of larval nutrition on nursing behavior. The red color indicates a diet of 150 µl (“undernourished”), the blue color that of 160 µl (“normal diet”), and the green color indicates 180 µl of food supply (“overfed”). Error bars indicate the 95% confidence interval in a conditional effects plot. Boxplots, with upper quartiles (75%) and lower (25%) represent the sampled data. Black dots indicate possible outliers and square boxes indicate jittered individual data points. **(A)** Larval nutrition did not significantly influence the probability of honeybees performing nursing tasks. Yet, the probability tended to decrease with increasing amounts of larval food. **(B)** Larval nutrition did not influence the onset of nursing. **(C)** Larval nutrition did not influence the termination of nursing. **(D)** Larval nutrition did not influence the nursing span. For test statistics and sample size, see **Tables 3**, **4** and **Supplementary Table 1**.

Larval nutrition did not show strong effects on nursing behavior (**Figure 1**; for detailed statistics see **Tables 3**, **4** and **Supplementary Table 1**). However, the probability of bees performing nursing tasks tended to decrease with increasing amounts of food (**Figure 1A**, predicted probability: 150 µl = 36%, 160 µl = 32%, 180 µl = 26%). No effects were observed for the onset of nursing (**Figure 1B**), the termination of nursing (**Figure 1C**) or the nursing span (**Figure 1D**).

**Figure 2:**
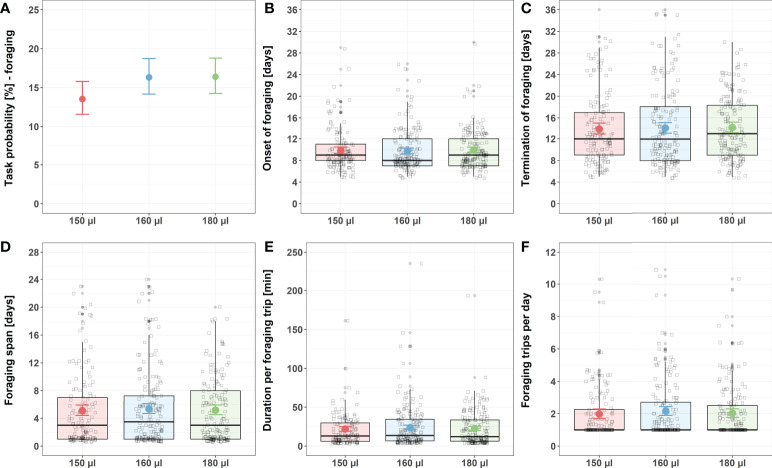
Influence of larval nutrition on foraging behavior. The red color indicates a diet of 150 µl (“undernourished”), the blue color that of 160 µl (“normal diet”), and the green color indicates 180 µl of food supply (“overfed”). Error bars indicate the 95% confidence interval in a conditional effects plot. Boxplots, with upper quartiles (75%) and lower (25%) represent the sampled data. Black dots indicate possible outliers and square boxes indicate jittered individual data points. **(A)** Larval nutrition did not significantly influence the probability of honeybees performing foraging tasks. **(B)** Larval nutrition did not influence the onset of foraging. **(C)** Larval nutrition did not influence the termination of foraging. **(D)** Larval nutrition did not influence the foraging span. **(E)** Larval nutrition did not influence the duration per foraging trip. **(F)** Larval nutrition did not influence the foraging trips per day. For test statistics and sample size, see **Tables 3**, **5**, **Supplementary Table 1**.

Larval nutrition did not affect foraging behavior (**Figure 2**; for detailed statistics see **Tables 3**–**5** and **Supplementary Table 2**). Different feeding regimes had no impact on the probability of bees performing foraging tasks (**Figure 2A**), the onset of foraging (**Figure 2B**), the termination of foraging (**Figure 2C**), the foraging span (**Figure 2D**), the duration of a foraging trip (**Figure 2E**) or the total number of foraging trips per day (**Figure 2F**).

### Weight, juvenile hormone titers, triglyceride levels and sucrose responsiveness

**Figure 3:**
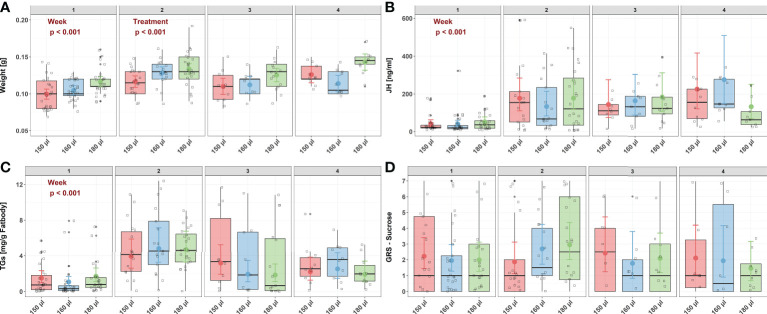
Influence of larval nutrition on the body weight, JH III titers, TG levels and sucrose responsiveness of adult honeybees in their first four weeks of life. The red color indicates a diet of 150 µl (“undernourished”), the blue color that of 160 µl (“normal diet”), and the green color indicates 180 µl of food supply (“overfed”). Error bars indicate the 95% confidence interval in a conditional effects plot. Boxplots, with upper quartiles (75%) and lower (25%) represent the sampled data. Black dots indicate possible outliers and square boxes indicate jittered individual data points. **(A)** Larval nutrition significantly influenced adult honeybee weight, with honeybees receiving less food being significantly lighter. However, weight increase with age. No significant interaction effect was found between age and diet. **(B)** JH levels were neither affected by diet nor by the interaction between age and diet. However, age significantly increased JH levels. **(C)** TG levels were neither affected by diet nor by the interaction between age and diet. However, TG levels increased in week two and decreased afterwards. **(D)** Neither age nor diet affected the sucrose responsiveness measured as gustatory response scores (GRS). For test statistics and sample sizes, see **Tables 7**, **6** and **Supplementary Tables 3, 4**.

Larval nutrition had a significant effect on the weight of adult honeybees (**Figure 3A**) but did not affect JH titers (**Figure 3B**), TG levels (**Figure 3C**) or the GRS (**Figure 3D**). For detailed statistics see **Tables 7**, **6** and **Supplementary Tables 3**, **4**. As assumed, undernourished honeybees (150 µl) weighed the least (predicted mean weight = 0.10 g) while overfed honeybees (180 µl) weighed the most (predicted mean weight = 0.12 g). Interestingly, honeybee weight increased significantly with age (**Figure 3A**, predicted mean weight week 1 – week 4: 0.10 g – 0.11 g) indicating a way for honeybees to compensate early food deprivation. However, the interaction between age and diet did not significantly influence honeybee weight (**Figure 3A**). As expected, JH levels were significantly influenced by age, with older honeybees showing higher JH levels then younger honeybees (**Figure 3B**, predicted mean JH titers week 1 – week 4: 41.53 ng/ml – 276 ng/ml). However, neither diet nor the interaction between age and diet significantly influenced JH levels (**Figure 3B**). Triglycerides were also affected by age. TG levels significantly increased in week 2 and decreased from then onwards (**Figure 3C**, predicted mean TG levels week w – week 4: 4.83 mg/g fat body – 2.53 mg/g fat body). However, neither diet nor the interaction between age and diet significantly influenced TG levels (**Figure 3C**). Sucrose responsiveness measured as GRS was not influenced by neither diet nor time (**Figure 3D**).

**Table 6:** Test statistics for the analysis conducted in **Figure 3**.

Analysis	Figure	Test	Week	Predicted	95% CIlower CI – upper CI
Weight [g]	3A	GLMM family = gaussian link = log	1 2 3 4	0.10 0.12 0.11 0.13	0.09 – 0.11 0.11 – 0.12 0.10 – 0.12 0.12 – 0.14
			ANOVA: χ = 52.20, **p < 0.001**		
JH [ng/ml]	3B	GLMM family = nbinom2 link = log	1 2 3 4	42.344 178 145 226	28.05 – 63-93 111 – 284 75.91 – 275 122 – 417
			ANOVA: χ = 112.086, **p < 0.001**		
TGs [mg/g]	3C	GLMM family = nbinom1 link = log	1 2 3 4	1.50 3.92 3.18 2.20	0.97 – 2.32 2.61 – 5.89 1.93 – 5.24 1.27 – 3.83
			ANOVA: χ = 80.068, **p < 0.001**		
GRS	3D	GLMM family = nbinom1 link = log	1 2 3 4	2.22 1.87 2.42 2.12	1.44 – 3.41 1.12 – 3.13 1.24 – 4.72 1.07 – 4.19
			ANOVA: χ = 2.358, p = 0.501		

Predicted means and the 95% confidence interval (CI) of the GLMM (including chosen family and link) analysis as well as the conducted ANOVA for weight, juvenile hormone (JH), triglycerides (TG) and the gustatory response score (GRS) in regard to the factor week. Results of the *post hoc* analysis are shown in **Supplementary Table 2**. Bold values indicate significant differences.

**Table 7:** Test statistics for the analysis conducted in **Figure 3**.

Analysis	Figure	Treatment	Sample size	Test	Predicted	95% CIlower CI – upper CI
Weight [g]	3A	150 µl 160 µl 180 µl	66 65 74	GLMM family = gaussian link = log	0.10 0.10 0.12	0.09 – 0.11 0.10 – 0.11 0.11 – 0.12
				ANOVA: χ = 33.74, **p < 0.001**		
JH [ng/ml]	3B	150 µl 160 µl 180 µl	61 65 72	GLMM family = nbinom2 link = log	4.23 4.15 5.27	2.80 – 6.39 2.80 – 6.16 3.51 – 7.91
				ANOVA: χ = 0.3992, p = 0.819		
TGs [mg/g]	3C	150 µl 160 µl 180 µl	65 64 74	GLMM family = nbinom1 link = log	1.5 1.07 1.7	0.971 – 2.32 0.683 – 1.68 1.1 – 2.62
				ANOVA: χ = 0.4142, p = 0.813		
GRS	3D	150 µl 160 µl 180 µl	66 65 74	GLMM family = nbinom1 link = log	2.22 1.95 1.99	1.44 – 3.41 1.27 – 2.97 1.27 – 3.12
				ANOVA: χ = 0.063, p = 0.969		

Predicted means and the 95% confidence interval (CI) of the GLMM (including chosen family and link) analysis as well as the conducted ANOVA for weight, juvenile hormone (JH), triglycerides (TG) and the gustatory response score (GRS) in regard to the factor treatment. Results of the *post hoc* analysis are shown in **Supplementary Table 2**. Bold values indicate significant differences.

## Discussion

In this study we reared honeybees *in-vitro* under different larval diets with larvae receiving 150 µl of food (“undernourished”), 160 µl of food (“normal diet”), or 180 µl of food (“overfed”). We expected severe effects of undernourishment on adult honeybees as it has been shown multiple times that poor nutrition can severely affect honeybee colonies, especially during autumn when the flowers stop blooming (57–59). As a reference point for undernourishment we used the standard artificial rearing diet which is known to be sufficient for honeybee rearing (30–33, 36). Earlier experiments showed effects of larval undernourishment on adult morphology, with undernourished larvae having slightly smaller thoraces and heads than honeybees reared under normal diet (37). Similar to that earlier study we found clear effects of larval diet on morphology. We show that diet significantly affected the weight of adult honeybees. Undernourished honeybees were the lightest and overfed honeybees weighed the most (**Figure 3A**) as shown before (27, 30, 37). However, the clear differences seem to disappear over the weeks when weight generally increased (**Supplementary Table 3**). Interestingly, undernourished honeybees tended to weigh even more then honeybees fed with the normal diet during the fourth experimental week (**Supplementary Table 4**). It seems that larval undernourishment, which correlates with a reduced growth, can be compensated for during adult development. Surprisingly, we found almost no effects of undernourishment on honeybee task performance and physiology, suggesting that physiology and behavior are not tightly linked to body weight and size.

Diet did not affect the proportion of honeybees performing either nursing or foraging tasks (**Figures 1A**, **2A**). However, we did observe a trend that honeybees receiving less food also had a higher chance of performing nursing tasks (**Table 3**, p = 0.0619). Yet, this tendency was lost when observing foraging proportion. This higher tendency to become a nurse bee might occur due to increased protein intake to compensate for undernourishment. Increased protein intake is known to increase VG levels (24), which are also increased in nurse bees (60). This further emphasizes the possible compensation of honeybees during adult maturation.

No differences were found in the foraging performance of undernourished or overfed honeybees in their foraging performance (**Figures 2B–F**). However, while they did not differ significantly in their foraging efficacy, honeybees might still differ in their foraging efficiency. Future studies should observe foraging intake to analyze whether undernourished honeybees transport the same amount of resources. Adult mortality is another interesting aspect which should be observed in future studies. While we measured the termination of foraging, it is not necessarily the same as mortality as mentioned above (Section 2.2). Undernourished honeybees may have returned to the hive for other tasks after they finished their foraging trips, although this phenomenon has not been reported to our knowledge. Therefore, they might have been less efficient compared to normally fed honeybees.

Juvenile hormone titers increased from the first week until the last week. These results indicate a typical transition from in-hive tasks to foraging, as JH titers are known to increase with age and tasks in typical honeybee colonies (14, 61). However, JH titers already increased in week two. Honeybees usually perform nursing tasks during their second week of adult life (6, 7). However, in our experiments, honeybees were kept in mini plus colonies which typically only hold about 3,000 to 5,000 honeybees (62), which may increase their maturation leading to increased JH titers. Contrary to our expectation, we did not observe an effect of diet on JH titers independent of age (**Figure 3B**). An earlier study showed that honeybees starved during larval development had increased JH hemolymph titers as adults compared to controls (27). However, starvation treatment was conducted different to our study. Wang etal. (27) reared honeybee larvae inside a normal colony and used pushing cages during the fifth larval instar to block nurse bees from feeding the larvae. Older larvae consume an increasing amount of food (30–33), leading to the conclusion that starvation during the fifth instar is more drastic than the undernourishment we applied during our *in-vitro* rearing.

Like JH, TG levels were not affected by the different diets but decreased significantly with age (**Figure 3C**) as shown before (11). As growing larvae need increasing amounts of food (30–33), starvation during the fifth instar leads to a strong reduction in larval food and therefore in proteins before pupation (57).

It is possible that slight undernourishment can be compensated for during early adult development. However, most cuticular structures cannot grow after they hardened (63). The observed gain in weight (**Figure 3A**) might not be a complete compensation and one needs to observe other morphological parameters in more detail. A first step would be to observe honeybee dry weight after undernourishment to differentiate between weight gain and water intake.

Furthermore, these results seem to indicate that starvation resistance of honeybees might be two-fold. In a recent study we showed that the *in-vitro* rearing protocol has a strong effect on adult honeybee task performance and physiology (36). Honeybees reared *in-vitro* performed significantly worse during foraging tasks and significantly fewer honeybees became foragers compared to hive reared controls. Interestingly, Scofield and Mattila (64) showed similar effects. Only 62% of honeybees became foragers when larvae were deprived of pollen, while about 82% of honeybees became foragers when they were raised with an abundance of pollen. They also showed that honeybees reared under pollen restricted conditions had an earlier foraging onset and terminated their foraging trips sooner than honeybees reared with an abundance of pollen (64). The similarity of the results between our earlier study (36) and the study by Scofield and Mattila (64) is striking. During the *in-vitro* rearing protocol, pollen is substituted with yeast. It seems likely that yeast does not contain the full composition of essential amino acids, essential lipids or essential sterols present in pollen (65–69). Lower quality protein sources might lead to lower quality bee bread as pollen is a major part of the bee bread fed to the larvae by the nurse bees (70). Therefore, *in-vitro* rearing (reduction in nutritional quality) significantly affects honeybees physiology and tasks performance while a reduction in nutritional quantity does not. Interestingly, Scofield and Mattila (64) showed significant effects of larval pollen deprivation on the weight of adult honeybee workers. Pollen deprived honeybees weighed significantly less compared to honeybees with an abundance of pollen, while honeybees reared under the standard *in-vitro* rearing protocol weighed as much as honeybees reared in the hive (36). This indicates that yeast supplement during *in-vitro* rearing supplies enough substance for growth but further emphasizes the possibility of missing essential amino acids, essential lipids or essential sterols during development. In conclusion, the reduced quantity of food supply during larval development appears not to lead to gross behavioral deficits, suggesting that honeybees are well buffered against this kind of nutritional stress. Honeybees seem to be able to compensate for short periods of larval undernourishment as long as they receive ample amounts of food as newly emerged adults.

## Data availability statement

The raw data supporting the conclusions of this article will be made available by the authors, without undue reservation.

## Author contributions

Conceptualization, RS and IS-D; methodology, FS, LH, and MK.; data analysis, FS, MK, LH, and MA; writing—original draft preparation, FS; writing—review and editing, FS, MK, MA, LH, RS, and IS-D; visualization, FS; supervision, RS, IS-D, and MM; software, FS and MA; project administration, IS-D, MM, and RS; funding acquisition, IS-D and RS. All authors have read and agreed to the published version of the manuscript.

## Funding

This study was supported by a grant of the Deutsche Forschungsgemeinschaft (DFG) through grants SCHE 1573/9-1 to RS and HA 5324/2-1 to IS-D and the projects 179877739 and 316629583. This publication was supported by the Open Access Publication Fund of the University of Wuerzburg.

## Acknowledgments

We thank our departmental beekeeper Dirk Ahrens. We thank Dr. Markus Thamm, Dr. Mira Becker, Fabienne Maihoff and Antonia Gommert for help during the larval rearing process. For metabolic analyses we thank the Metabolomics Core Unit of the University Würzburg. We also thank the reviewers for their great advice regarding the manuscript.

## Conflict of interest

The authors declare that the research was conducted in the absence of any commercial or financial relationships that could be construed as a potential conflict of interest.

## Publisher’s note

All claims expressed in this article are solely those of the authors and do not necessarily represent those of their affiliated organizations, or those of the publisher, the editors and the reviewers. Any product that may be evaluated in this article, or claim that may be made by its manufacturer, is not guaranteed or endorsed by the publisher.
