# Corrigendum: Honeybees are buffered against undernourishment during larval stages

**DOI:** 10.3389/finsc.2023.1146464

**Published:** 2023-01-31

**Authors:** Felix Schilcher, Lioba Hilsmann, Markus J. Ankenbrand, Markus Krischke, Martin J. Mueller, Ingolf Steffan-Dewenter, Ricarda Scheiner

**Affiliations:** ^1^ Behavioral Physiology and Sociobiology, Theodor-Boveri-Institute, Biocenter, Julius-Maximilians- Universität Würzburg, Würzburg, Germany; ^2^ Center for Computational and Theoretical Biology (CCTB), Julius-Maximilians-Universität Würzburg, Würzburg, Germany; ^3^ Julius-von-Sachs-Institute of Biosciences, Theodor-Boveri-Institute, Biocenter, Julius-Maximilians-Universität Würzburg, Würzburg, Germany; ^4^ Animal Ecology and Tropical Biology, Theodor-Boveri-Institute, Biocenter, Julius-Maximilians-Universität Würzburg, Würzburg, Germany

**Keywords:** nutrition, *in-vitro* rearing, juvenile hormone, nurse bees, foragers, triglycerides, undernourishment, task allocation

In the published article, there was an error in the Funding statement. Funding support from the Open Access Publication Fund of the University of Wuerzburg was erroneously excluded. The correct Funding statement appears below.

This study was supported by a grant of the Deutsche Forschungsgemeinschaft (DFG) through grants SCHE 1573/9-1 to RS and HA 5324/2-1 to IS-D and the projects 179877739 and 316629583. This publication was supported by the Open Access Publication Fund of the University of Wuerzburg.

The authors apologize for this error and state that this does not change the scientific conclusions of the article in any way. The original article has been updated.

