# Supplementary material for: Honeybees are buffered against undernourishment during larval stages
Source: Front Insect Sci. 2022 Nov 18;2:951317. doi: 10.3389/finsc.2022.951317 (PMC10926507; doi:10.3389/finsc.2022.951317)
Supplement: Supplementary file 1 [file DataSheet_1.docx]

Supplementary Material

# Supplementary Figures and Tables

## Supplementary Figures


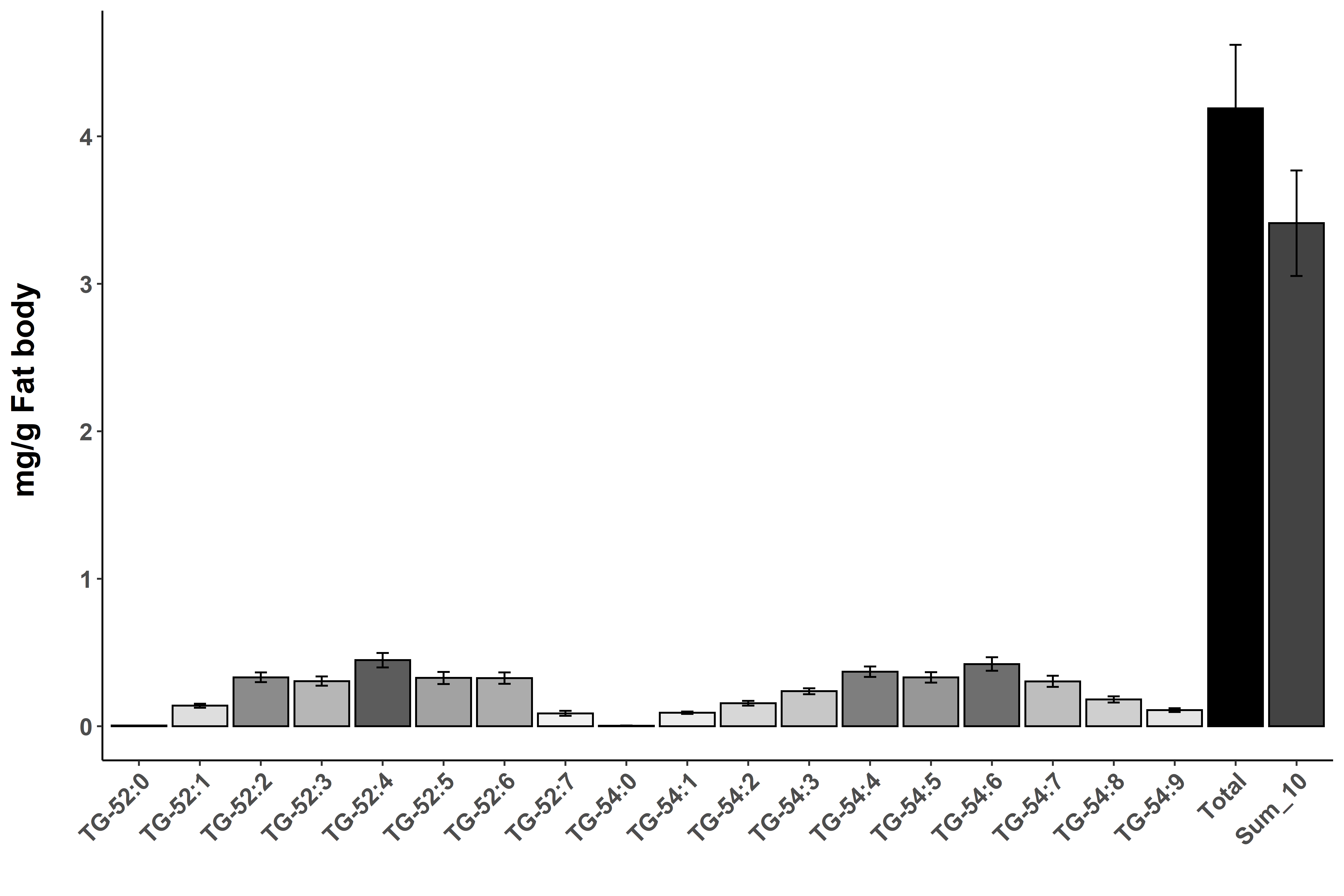


**Supplementary Figure 1.** Total amount of Triglycerides, sum of the ten most frequently appearing TGs and all TGs measurable in the honeybee fat body of 130 honeybees. The sum of the ten most frequently appearing TGs is equal to approx. 80% of the sum of all measured TGs in the honeybee fat body. The darker the color, the more mg TGs / g fat body were present. Error bars show the standard error.

Supplementary Table 1: Tukey post hoc tests for the analysis conducted in Figure 1 and Table 4.

| **Analysis** | **Figure** | **Contrasts** | **Odds ratio** | **Significance** |
| --- | --- | --- | --- | --- |
| Onset of nursing | 1B | 150 µl vs 160 µl  150 µl vs 180 µl  160 µl vs 180 µl | 0.970  0.957  0.987 | p =0.8247  p = 0.7176  p = 0.9722 |
| Termination of nursing | 1C | 150 µl vs 160 µl  150 µl vs 180 µl  160 µl vs 180 µl | 0.927  0.960  1.036 | p = 0.3319  p = 0.7711  p = 0.8230 |
| Nursing span | 1D | 150 µl vs 160 µl  150 µl vs 180 µl  160 µl vs 180 µl | 0.838  0.928  1.107 | p = 0.4395  p = 0.8933  p = 0.7991 |

Supplementary Table 2: Tukey post hoc tests for the analysis conducted in Figure 2 and Table 5.

| **Analysis** | **Figure** | **Contrasts** | **Odds ratio** | **Significance** |
| --- | --- | --- | --- | --- |
| Onset of foraging | 2B | 150 µl vs 160 µl  150 µl vs 180 µl  160 µl vs 180 µl | 1.01  0.994  0.983 | p =0.9690  p = 0.9871  p = 0.9102 |
| Termination of foraging | 2C | 150 µl vs 160 µl  150 µl vs 180 µl  160 µl vs 180 µl | 0.993  0.986  0.993 | p = 0.9905  p = 0.9618  p = 0.9893 |
| Foraging span | 2D | 150 µl vs 160 µl  150 µl vs 180 µl  160 µl vs 180 µl | 0.952  0.986  1.036 | p = 0.8687  p = 0.9890  p = 0.9216 |
| Duration per foraging trip | 2E | 150 µl vs 160 µl  150 µl vs 180 µl  160 µl vs 180 µl | 0.921  0.970  1.053 | p = 0.5995  p = 0.9336  p = 0.7944 |
| Foraging trips per day | 2F | 150 µl vs 160 µl  150 µl vs 180 µl  160 µl vs 180 µl | 0.960  0.983  1.024 | p = 0.8270  p = 0.9671  p = 0.9306 |

Supplementary Table 3: Tukey post hoc tests for the analysis conducted in Figure 3 and Table 6, 7

| **Analysis** | **Figure** | **Contrasts** | **Odds ratio** | **Significance** |
| --- | --- | --- | --- | --- |
| Weight  Factor Week | 3A | Week 1 vs Week 2  Week 1 vs Week 3  Week 1 vs Week 4  Week 2 vs Week 3  Week 2 vs Week 4  Week 3 vs Week 4 | 0.847  0.922  0.839  1.089  0.991  0.910 | **p < 0.001**  p = 0.08  **p < 0.001**  **p = 0.048**  p = 0.97  **p = 0.047** |
| Weight  Factor Treatment | 3A | 150 µl vs 160 µl  150 µl vs 180 µl  160 µl vs 180 µl | 0.984  0.871  0.886 | p = 0.8450  **p < 0.001**  **p < 0.001** |
| JH  Factor Week | 3B | Week 1 vs Week 2  Week 1 vs Week 3  Week 1 vs Week 4  Week 2 vs Week 3  Week 2 vs Week 4  Week 3 vs Week 4 | 0.279  0.277  0.224  0.991  0.801  0.808 | **p < 0.001**  **p < 0.001**  **p < 0.001**  p = 0.999  p = 0.754  p = 0.7769 |
| JH  Factor Treatment | 3B | 150 µl vs 160 µl  150 µl vs 180 µl  160 µl vs 180 µl | 0.993  1.016  1.024 | p = 0.999  p = 0.995  p = 0.988 |
| TGs  Factor Week | 3C | Week 1 vs Week 2  Week 1 vs Week 3  Week 1 vs Week 4  Week 2 vs Week 3  Week 2 vs Week 4  Week 3 vs Week 4 | 0.313  0.621  0.635  1.986  2.028  1.021 | **p < 0.001**  **p = 0.025**  p = 0.059  **p < 0.001**  **p < 0.001**  p = 0.999 |
| TGs  Factor Treatment | 3C | 150 µl vs 160 µl  150 µl vs 180 µl  160 µl vs 180 µl | 1.130  1.101  0.974 | p = 0.660  p = 0.766  p = 0.9818 |
| GRS  Factor Week | 3D | Week 1 vs Week 2  Week 1 vs Week 3  Week 1 vs Week 4  Week 2 vs Week 3  Week 2 vs Week 4  Week 3 vs Week 4 | 0.832  0.984  1.127  1.182  1.355  1.146 | p = 0.745  p = 0.999  p = 0.964  P = 0.893  p = 0.629  p = 0.966 |
| GRS  Factor Treatment | 3D | 150 µl vs 160 µl  150 µl vs 180 µl  160 µl vs 180 µl | 1.04  1.04  1.00 | p = 0.982  p = 0.978  p = 0.999 |

Supplementary Table 4: Predicted mean values and the 95% confidence interval (CI) of the GLMM analysis for weight, juvenile hormone (JH), triglycerides (TGs), and the gustatory response score (GRS) in Figure 3.

|  | 150 µl | | | 160 µl | | | 180 µl | | |
| --- | --- | --- | --- | --- | --- | --- | --- | --- | --- |
| Analysis | Week | Predicted | 95% CI  Lower CI – upper CI | Week | Predicted | 95% CI  Lower CI – upper CI | Week | Predicted | 95% CI  Lower CI – upper CI |
| Weight  [g] | 1 | 0.10 | 0.09 – 0.11 | 1 | 0.10 | 0.10 – 0.11 | 1 | 0.12 | 0.11 – 0.12 |
|  | 2 | 0.12 | 0.11 – 0.12 | 2 | 0.13 | 0.12 – 0.14 | 2 | 0.13 | 0.13 – 0.14 |
|  | 3 | 0.11 | 0.10 – 0.12 | 3 | 0.11 | 0.10 – 0.12 | 3 | 0.13 | 0.12 – 0.13 |
|  | 4 | 0.13 | 0.12 – 0.14 | 4 | 0.11 | 0.10 – 0.12 | 4 | 0.14 | 0.13 – 0.15 |
| Interaction  Treatment & Week | | | GLMM  family = gaussian  link = log | | χ = 7.8177 | p = 0.2518 |  |  |  |
| JH [ng/ml] | 1 | 42.34 | 28.05 – 63.93 | 1 | 41.53 | 28.02 – 61.55 | 1 | 52.72 | 35.12 – 79.15 |
|  | 2 | 178 | 111 – 284 | 2 | 134 | 84.25 – 214 | 2 | 178 | 118 – 269 |
|  | 3 | 145 | 75.91 – 275 | 3 | 164 | 89.09 – 304 | 3 | 184 | 108 – 312 |
|  | 4 | 226 | 122 - 417 | 4 | 276 | 149 - 509 | 4 | 133 | 70.49 - 252 |
| Interaction  Treatment & Week | | | GLMM  family = nbinom2  link = log | | χ = 6.4584 | p = 0.3711 |  |  |  |
| TGs  [mg/g] | 1 | 1.5 | 0.97 – 2.32 | 1 | 1.07 | 0.68 – 1.68 | 1 | 1.7 | 1.1 – 2.62 |
|  | 2 | 3.92 | 2.61 – 5.89 | 2 | 4.83 | 3.26 – 7.15 | 2 | 4.7 | 3.27 – 6.76 |
|  | 3 | 3.18 | 1.93 – 5.24 | 3 | 1.94 | 1.08 – 3.47 | 3 | 1.84 | 1.1 – 3.07 |
|  | 4 | 2.2 | 1.27 – 3.83 | 4 | 2.53 | 1.48 – 4.33 | 4 | 1.92 | 1.08 – 3.39 |
| Interaction  Treatment & Week | | | GLMM  family = nbinom1  link = log | | χ = 9.0324 | p = 0.1718 |  |  |  |
| GRS | 1 | 2.22 | 1.44 – 3.41 | 1 | 1.95 | 1.27 – 2.97 | 1 | 1.99 | 1.27 – 3.12 |
|  | 2 | 1.87 | 1.12 – 3.13 | 2 | 2.70 | 1.72 – 4.25 | 2 | 2.96 | 2.00 – 4.37 |
|  | 3 | 2.42 | 1.24 – 4.72 | 3 | 1.78 | 0.84 – 3.79 | 3 | 2.10 | 1.19 – 3.69 |
|  | 4 | 2.12 | 1.07 – 4.19 | 4 | 1.94 | 0.91 – 4.16 | 4 | 1.46 | 0.68 – 3.17 |
| Interaction  Treatment & Week | | | GLMM  family = nbinom1  link = log | | χ = 3.0965 | p = 0.7966 |  |  |  |
